# Recursive sequence generation in crows

**DOI:** 10.1126/sciadv.abq3356

**Published:** 2022-11-02

**Authors:** Diana A. Liao, Katharina F. Brecht, Melissa Johnston, Andreas Nieder

**Affiliations:** Animal Physiology, Institute of Neurobiology, University of Tübingen, Auf der Morgenstelle 28, Tübingen 72076, Germany.

## Abstract

Recursion, the process of embedding structures within similar structures, is often considered a foundation of symbolic competence and a uniquely human capability. To understand its evolution, we can study the recursive aptitudes of nonhuman animals. We adopted the behavioral protocol of a recent study demonstrating that humans and nonhuman primates grasp recursion. We presented sequences of bracket pair stimuli (e.g., [ ] and { }) to crows who were instructed to peck at training lists. They were then tested on their ability to transfer center-embedded structure to never-before-seen pairings of brackets. We reveal that crows have recursive capacities; they perform on par with children and even outperform macaques. The crows continued to produce recursive sequences after extending to longer and thus deeper embeddings. These results demonstrate that recursive capabilities are not limited to the primate genealogy and may have occurred separately from or before human symbolic competence in different animal taxa.

## Teaser

Crows can learn to produce demanding center-embedded recursive bracket sequences, e.g., {[( )]}.

## INTRODUCTION

Recursion, the cognitive capacity to embed an element structure within others of the same kind, has been claimed as one of the key features of human symbolic competence (*1*). It has been put forth as what distinguishes human language from all other forms of animal communication (*2*) to which counter-arguments have been made [see (*3*)]. Grammatical rules in language use recursion to expand the variety and complexity of possible sentences that could be produced into what is conceptually infinite. In the Chomsky hierarchy of grammars with increasing generative power, center-embedded recursion is said to sit near the top, below context-sensitive (and Turing-complete) grammars. In this grammar, equivalent procedures are embedded in the middle of a sequence. One of the classic example sentences with such a center-embedded structure is “The mouse (A^1^) the cat (A^2^) chased (B^2^) ran (B^1^)”. Here, the inner clause “the cat (A^2^) chased (B^2^)” is embedded within the outer clause “the mouse (A^1^) [that] ran (B^1^)”. Such expressions can be formalized as A^n^B^n^, a context-free grammar.

To understand the evolution of symbolic skills, there has been considerable interest in investigating whether nonhuman animals can perceive and produce recursive sequences. Several prominent studies have explored differences in animals’ auditory perception of finite-state grammars, i.e., (AB)^n^, and context-free grammars, i.e., A^n^B^n^ (*4*–*6*). However, these results have been controversial given that alternative, nonrecursive strategies for task performance have been put forward [e.g., such as discriminating different numbers of syllables; (*7*, *8*)]. The stimuli used (e.g., different syllable types) are not intrinsically paired, such that that A^1^-B^1^ is not differentiated from A^2^-B^2^. This lack of inherent relationship between stimuli pairs allows for certain exploits that superficially appear like recursive responses but can be explained with simpler phonetic or numerical strategies (*9*–*11*). Therefore, for the demonstration of any context-free grammar, semantic pairing is of paramount importance. However, separate training on the associations between pairs of arbitrary elements could introduce potential bias to test responses once pairs are combined into longer sequences (*12*, *13*).

**Fig. 1.**
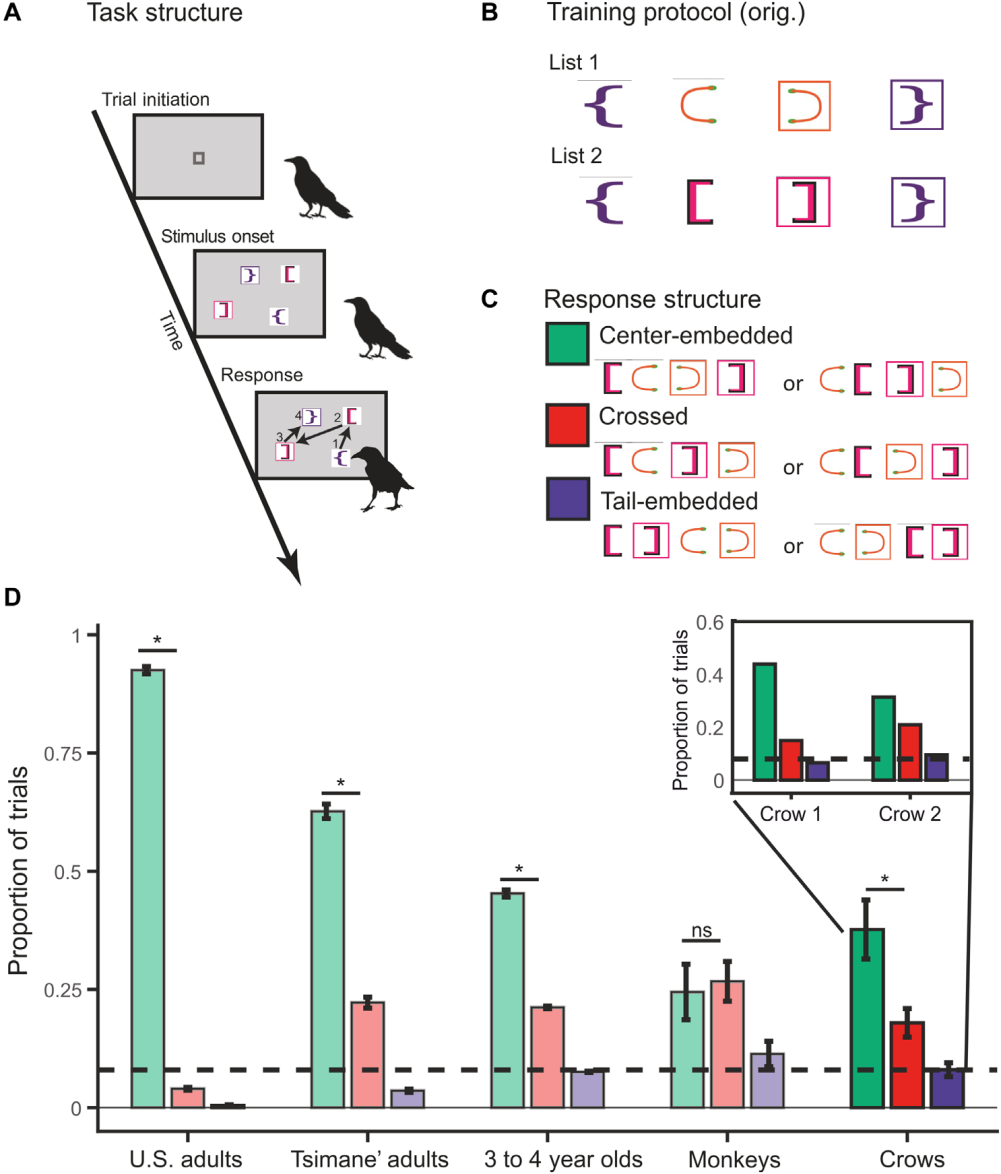
Bracket task design and performance on transfer trials for crows. (**A**) Training procedure: Crows were required to peck at the bracket stimuli in a center-embedded sequence order. After initiating a trial, two pairs of brackets appeared simultaneously in random locations on the touchscreen monitor. The crow was required to peck each stimulus in a determined order (depicted here with arrows and numbers) and was rewarded for correct sequence completion. Otherwise, when an incorrect bracket was selected, the screen flashed, an error tone played, and brief time-out was initiated. (**B**) Training lists that were presented until criterion was reached. After training, these lists were intermixed within a session along with transfer trials that consisted of the inner bracket pairs from the two training lists. (**C**) Color-coded main response types to the transfer trials—derived from the training lists—which were rewarded regardless of the selected order. (**D**) Proportion of response types produced by the crows (in saturated colors) as compared to U.S. adults, Tsimane’ adults, U.S. children, and monkeys [in faded colors; (*14*)]. Response strategies color-coded as in (C). Inset displays results for each crow. Error bars represent the SEM of the population, “*” represents a significant difference (*P* < 0.05) between the proportion of center-embedded and crossed responses, and ns represents no significant difference between groups. Bracket stimuli modified from (*14*) @ The Authors, some rights reserved; exclusive licensee *The American Association for the Advancement of Science*. Distributed under a CC BY-NC 4.0 license (http://creativecommons.org/licenses/by-nc/4.0/).

A recent study (*14*) cleverly addressed these issues. They introduced different pairs of colored brackets that are intrinsically linked with an open and closed direction (e.g., [ ], { }, and < > ; Fig. 1, B and C). Using these innovative stimuli in experiments with U.S. adults, Tsimane’ adults, U.S. children, and rhesus monkeys (*Macaca mulatta*), functionally equivalent training and testing procedures were performed for all groups so that results could be directly compared. Participants were trained to first touch one of the training sequences, e.g., { ( ) } and { [ ] }, in these specific orders. They were then tested on the ability to spontaneously transfer this recursive, center-embedded structure to the novel pairing of bracket sequences, e.g., ( [ ] ) or [ ( ) ]. All humans were able to successfully complete this test, and the monkeys, with additional training, did so as well. These results demonstrate that primates across age, education, culture, and species could all learn to produce basic recursive sequences with nested pairs of bracket stimuli.

Because recursive sequence generation applying this bracket protocol has been tested exclusively in primate species so far, the implied assumption is that the ability to track syntactic relationships between elements over distances may have been inherited from a common primate ancestor before reaching its most elaborate and specific expression in humans (*15*). Inspired by the recent study (*14*), we replicate and extend the bracket protocol in crows (*Corvus corone*) to examine recursive behavior through a comparative evolutionary lens. Crows are corvids, a songbird family that exhibit complex cognition such as elaborate tool use (*16*–*18*), analogical reasoning (*19*), rule switching (*20*), and numerical competency (*21*, *22*). As songbirds (*23*), their vocal communication skills show interesting parallels with human speech, such as complex acoustic signals, sensitive learning periods, the need for auditory feedback, elaborate vocal production abilities, and social learning (*24*–*26*). Combined, these traits make crows a promising candidate to search for an understanding of recursive primitives.

## RESULTS

### Center-embedded sequence generation in crows (experiment 1)

Mirroring the macaque monkey training procedures (*14*), two carrion crows were presented with four brackets displayed at random positions on a touchscreen monitor. They would then be rewarded for pecking the brackets in a specific order (Fig. 1A). These colored brackets are composed of symmetric pairs with one bracket each facing an open and closed direction; thus, they were intrinsically linked. For the two bracket pairs { } and ( ), for instance, the crows were trained to peck the sequence “{ ( ) }”. Crows first had to master two different bracket lists, list 1 and list 2 (Fig. 1B). These lists contained a center-embedded structure but did not necessarily need to be represented as such to succeed in the task. After reaching criterion on both lists (>70% accuracy over 2 days; chance ~4%; see table S1 for total training trials until criterion), the crows were presented with transfer trials—at a frequency of 10% interleaved with the training lists—to examine whether they generalized the recursive center-embedded structure. The transfer trials consisted of novel combinations of the inner two bracket pairs derived from the two training lists (i.e., “[ ]” and “( )”) that were never shown together during training (Fig. 1C). On the transfer trials, crows received a reward for pecking in any order as long as four pecks were registered, thus precluding learning about any specific response structure during testing. Therefore, the transfer trials served to investigate how these sequences were mentally represented by the crows.

Similar to humans and primates, most of the transfer trial responses fell into three main response structures (fig. S1): tail-embedded responses, nonrecursive crossed responses, and recursive center-embedded responses. The proportions with which these response structures are produced could reflect the potential strategy adopted by the crows to solve the task. First, if the crows were using an associative chain strategy whereby the subject maximizes familiar orderings between pairs from preceding training trials, then we would expect a high proportion of tail-embedded responses “[ ] ( )” or “( ) [ ]” (Fig. 1C). We found that both crows used this strategy not significantly different from chance level (17 of 212, ~8%; crow 1: 6.54%; crow 2: 9.52%; two-tailed binomial test, *P* = 0.595; Fig. 1D). Thus, this associative chain strategy could therefore be excluded.

Next, if the crows learned that certain sequence positions were equally represented during training, then we would expect an equal proportion of crossed responses “[ ( ] )” or “( [ ) ]” and center-embedded responses “( [ ] )” or “[ ( ) ]”. Specifically, the crows could have abstracted the nonrecursive pattern that open brackets were positioned in the first half of the sequence and closed brackets positioned in the later half (Fig. 1C). If this strategy was adopted, then closed brackets would be chosen in the latter half of the sequence, regardless of whether that bracket would match the most recently selected open bracket. However, we found that crows applied crossed responses during ~20% of the transfer trials (crow 1: 15.0%; crow 2: 20.9%; Fig. 1D). In addition, the frequency of response patterns was not equally distributed [chi-square test of goodness of fit: χ^2^ (*df* = 2, *N* = 135) = 45.73, *P* < 0.001]. We found that the crows produced significantly less crossed responses than center-embedded responses [38 versus 118, chi-square test of goodness of fit: χ^2^ (*df* = 1, *N* = 118) = 14.95, *P* = 0.002; significant at Bonferroni-corrected α = 0.05/2]; the ordinal strategy therefore could also not account for the general response behavior of the crows.

The production of center-embedded responses structure indicates the understanding that a matched opened and closed bracket pair is embedded in the middle of another opened and closed bracket pair (Fig. 1C). We found that the center-embedded response structure was the most preferred among the three main response structures and was used in about 40% of the transfer trials (crow 1: 43.9%; crow 2: 31.4%; Fig. 1D). This performance was significantly above chance (80 of 212, two-tailed binomial test, *P* < 0.001) and above the proportion of other major response categories (chi-square test of independence: χ^2^ = 45.73, *P* < 0.001). The crows’ proportion of center-embedded responses was not significantly different from that of human children but was higher than the proportion of center-embedded responses of the monkeys [chi-square test between children and crows: χ^2^ (*df* = 1, *N* = 419) = 0.56, *P* = 0.453, chi-square test between monkeys and crows: χ^2^ (*df* = 1, *N* = 315) = 7.46, *P* = 0.006, significant at Bonferroni-corrected α = 0.05/3; Fig. 1D] (*14*). Recursive responses for both crows in experiment 1 were robust throughout the course of testing (table S2). These results show that crows outperformed the monkeys while demonstrating a similar performance to children. With additional training with two novel bracket lists, monkeys were able to start producing more center-embedded than crossed responses. We also performed a chi-square test between the current crow results (first exposure) and the monkey responses with further training (second exposure). We find no significant difference (χ^2^ = 0.1955, *P* = 0.658). Thus, crows join humans in being able to spontaneously represent and transfer a center-embedded recursive structure given only few exemplars.

### Training position affects center-embedded sequence generation (experiment 2)

**Fig. 2.**
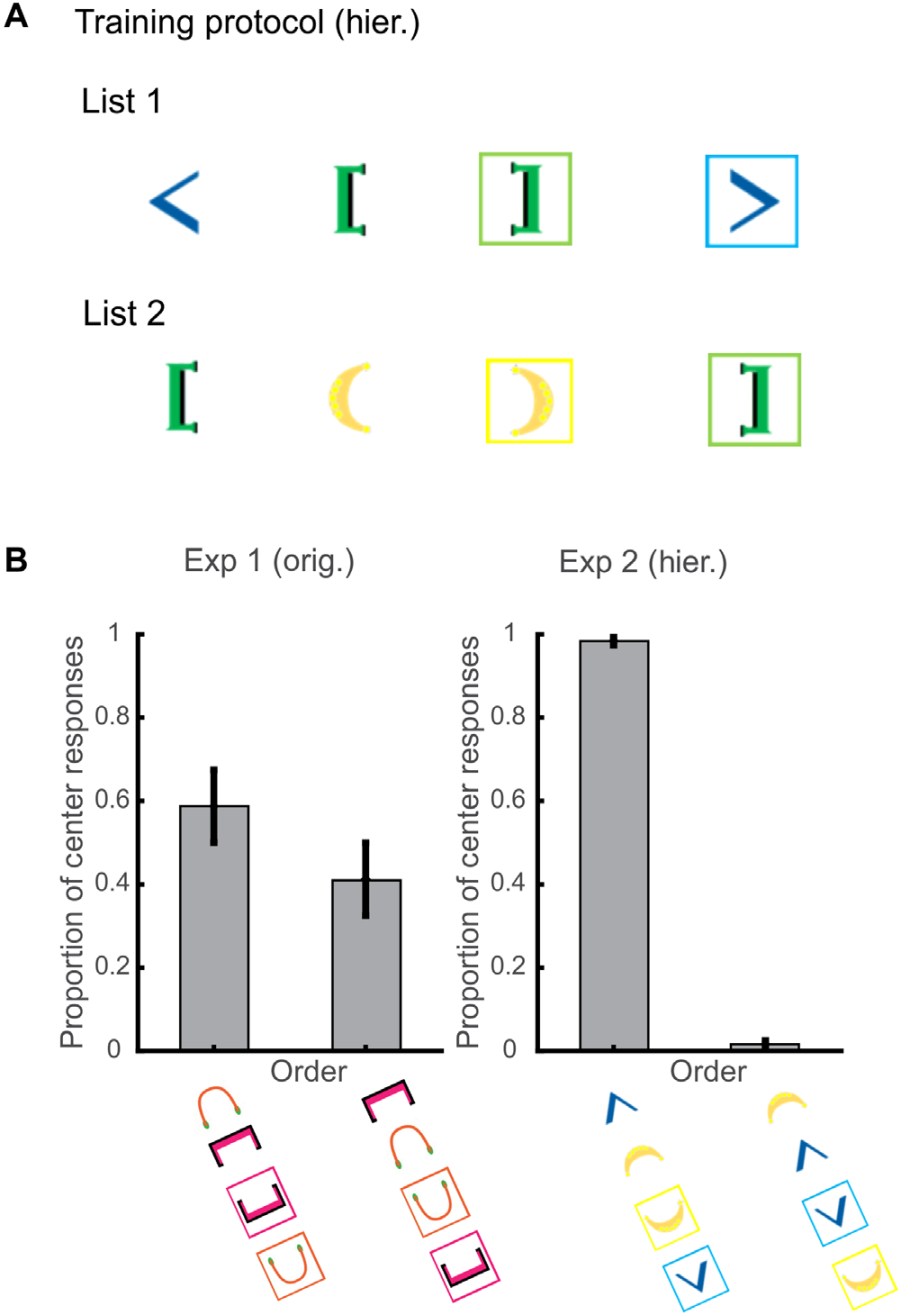
Hierarchical ordering of bracket stimuli. (**A**) Following identical procedures of experiment 1, two new training lists were presented until criterion was reached. Again, transfer trials were introduced that consisted of one unique pair of brackets from each of the training lists. (**B**) Valid center-embedded responses with the two pairs can be ordered in two different ways. In experiment 1, there was no difference in the order. In this experiment, test pairs were composed of the outer pair from list 1 and the inner pair from list 2. The crows vastly preferred responding in an outside-to-in pair manner when producing center-embedded sequences.

Next, we explored how the positioning of the training stimuli affects responses during transfer trials. We wanted to test whether the crows realized that elements are bound from outside to in; therefore, we modified the training procedures accordingly. Instead of presenting a consistent bracket pair as the outer pair in both training lists while changing the inner pair identities, the consistent pair “[ ]” in green was the inner pair on one list and the outer pair on the other (Fig. 2A). For list 1, “< >” blue brackets served as the outer pair, while “( )” yellow brackets served as the inner pair for list 2. During transfer trials in which the “< >” and “( )” brackets were presented together, the hierarchical order of the brackets could be inferred on the basis of their relationship to the “[ ]” brackets (see table S1 for total training trials until criterion). Examining the response patterns (fig. S2) for these transfer trials revealed that the crows produced more center-embedded responses than expected by chance (two-tailed binomial test, 184 of 253, *P* < 0.001). The frequency of response patterns was not equally distributed [chi-square test of goodness of fit: χ^2^ (*df* = 2, *N* = 205) = 293.86, *P* < 0.001]. Again, the crows produced significantly less crossed responses than center-embedded responses [13 versus 184, chi-square test of goodness of fit: χ^2^ (*df* = 1, *N* = 197) = 184.43, *P* < 0.001; significant at Bonferroni-corrected α = 0.05/2]. Recursive responses for both crows in experiment 2 were robust throughout the course of testing (table S2). Thus, the results of experiment 2 mirror and corroborate the results from experiment 1.

With only two pairs used in transfer trials, we had two potential response orders (Figs. 1C and 2B) that both fulfill a center-embedded response structure (fig. S2). Notably, there was a significant association between order and experiment [chi-square test of independence: χ^2^ (*df* = 1, *N* = 265) = 74.76, *P* < 0.001]: In experiment 1, where both inner pairs were presented together, we found that there was no significant difference in the frequency with which the orders were produced [( [ ] ): 47 of 80, [ ( ) ]: 33 of 80; chi-square test of goodness of fit: χ^2^ (*df* = 1, *N* = 80) = 2.45, *P* = 0.118; Fig. 2B]. However, in experiment 2, where one outer pair “< >” and one inner pair “( )” were presented together, the crows consistently preferred responding in this bounded manner [< () >: 182 of 185, ( < > ): 3 of 185: chi-square test of goodness of fit: χ^2^ (*df* = 1, *N* = 185) = 176.09, *P* < 0.001, significant at Bonferroni-corrected α = 0.05/3; Fig. 2B]. This result suggests that the crows may be sensitive to the hierarchical bounded structure of the center-embedded sequences.

### Sequences of deeper embedding (experiment 3)

While experiment 2 hinted that crows were sensitive to the relative ordering of stimuli, with only two pairs of brackets, it cannot be excluded that they simply used an ordinal strategy to encode identity and position for those lists. To address this possibility, we next made the sequences more challenging by increasing recursive depth. This increased length also allows us to examine brackets in which the hierarchical ordering and the original training positions are in conflict. In principle, recursion is an infinite combinatorial capacity, only limited by working memory capacity. We therefore examined whether the crows were able to retain their ability to represent center-embedded sequences when challenged by longer and thus more demanding sequences.

**Fig. 3.**
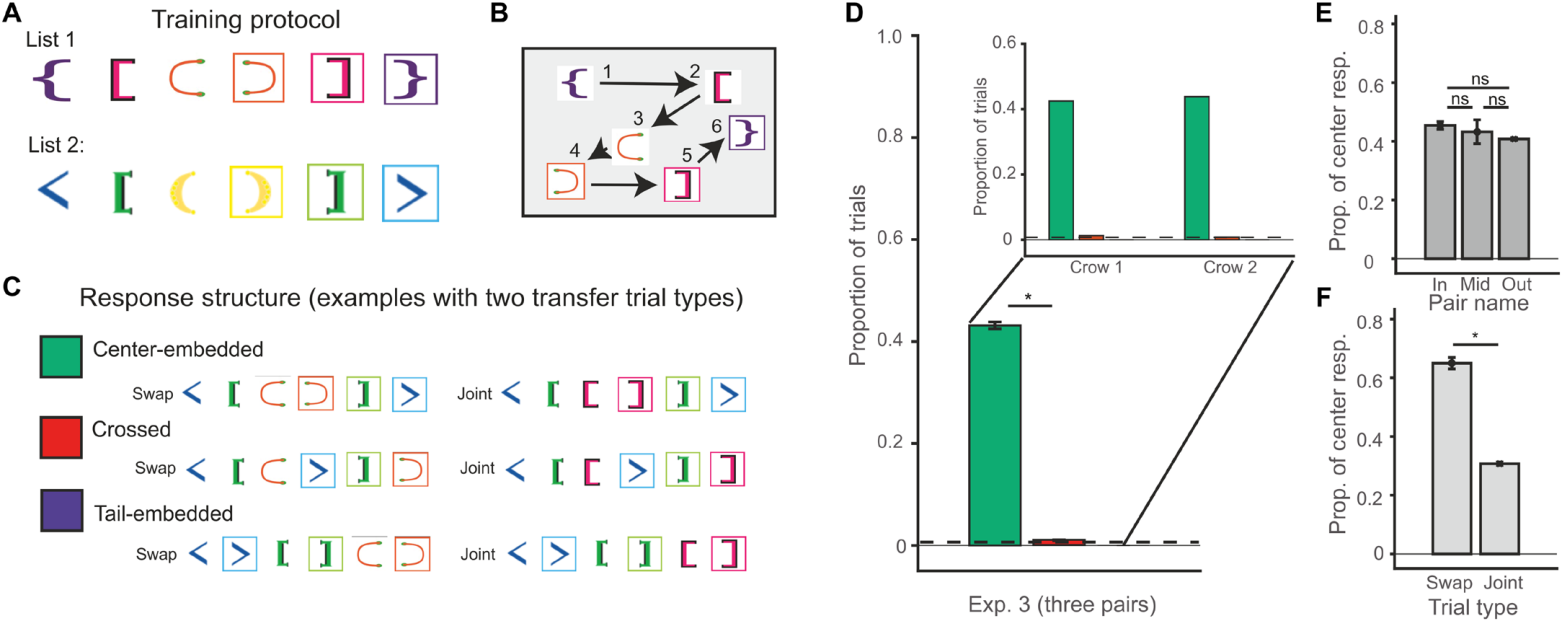
Three pairs of brackets stimuli task design and performance. (**A**) Two new training lists consisting of three pairs of brackets were presented during training for experiment 3. After training, these lists were intermixed along with a small proportion of transfer trials. Transfer trials were constructed from every combination of the training list pairs (see fig. S3). (**B**) Example of a three-pair training trial on screen with arrows/numbers indicating an order pecked to generate a valid center-embedded recursive sequence. (**C**) Examples of response structures categorized into two types of transfer trials (swap and joint). In swap trials, pairs of the same position in each list were switched (i.e., the orange inner pair of training list 1 replaced the yellow inner pair of training list 2). In joint trials, pairs of the same position in each training list were presented together (i.e., the pink middle pair of training list 1 replaced the yellow inner pair of training list 2 such that there were two middle pairs in the new test list). (**D**) Proportion of response types produced. Responses are color-coded according to response types in (C); inset displays results for each crow. (**E**) Proportion of center responses produced separated by position of transfer trial pair types. (**F**) Proportion of center responses produced separated by transfer trial combination types. Error bars represent the SEM of the population, “*” represents a significant difference (*P* < 0.05), and ns represent no significant difference between groups.

Thus, we presented the crows with an increased depth of the bracket sequences of three pairs. We constructed two training lists of three bracket pairs from the pairs used in experiments 1 and 2 (Fig. 3A). Once the crows mastered each of the training lists (60% accuracy over 2 days, chance ~0.01%; see table S1 for total training trials until criterion), they were given test sessions with transfer trials. Sessions consisted of 90% intermixed trials from the two training lists and 10% novel transfer trials (fig. S3). There were 18 possible stimulus combinations for the transfer lists. We alternatingly chose six transfer lists per session and categorized them according to which pair positions were manipulated (i.e., inner, middle, or outer positions). We also separated the transfer lists into two potential trial types: swap trials, where pairs of the same position were switched onto the opposing lists (e.g., a list where two inner pairs were switched); and joint trials, where pairs of the same position were placed together (e.g., a list composed of one outer pair and two inner pairs) (Fig. 3B). As with previous transfer trials, crows received a reward for pecking in any order as long as six pecks were registered.

Responses were assigned to the three categories used for the two pair experiments (i.e., tail-embedded responses, nonrecursive crossed responses, and recursive center-embedded responses; Fig. 3C). We found that the crows produced no tail-embedded responses. In addition, the crows barely produced crossed responses (crow 1: 1.3%; crow 2: 0.8%). The majority of responses by far were center-embedded sequences, with a proportion of 42.5 and 43.8% for crow 1 and crow 2, respectively. Both crows followed this recursive center-embedded strategy far more often than expected by chance (579 of 1343; two-tailed binomial test; *P* < 0.001). The frequency of response pattern was not equally distributed [chi-square test of goodness of fit: χ^2^ (*df* = 2, *N* = 593) = 1104, *P* < 0.001]. The crows also produced significantly more center-embedded responses compared to crossed responses [579 of 593 responses were center-embedded; chi-square test of goodness of fit: χ^2^ (*df* = 1, *N* = 593) = 538.32, *P* < 0.001, significant at Bonferroni-corrected α = 0.05/3]. Again, recursive responses for both crows in experiment 3 were robust throughout the course of testing (table S2).

To examine whether the position of the pairs involved in the transfer trials affected performance, we examined response proportions for the inner, middle, and outer pair position trials separately (Fig. 3D). We found no significant difference in the trials separated by pair position (inner: 45.5%, middle: 42.9%, outer: 40.8%; two-sided Fisher’s exact test due to empty cells, *P* = 0.410). Last, we split the trials into their respective “swap” or “joint” categories to examine whether there were differences in this performance (swap: 313 of 482 and joint: 266 of 863). Both trial types were responded to in a center-embedded manner well above chance (two-tailed binomial test: swap *P* < 0.001; joint *P* < 0.001), albeit that trial type influences the frequency of the respective response patterns [chi-square test of independence: χ^2^ (*df* = 1, *N* = 1345) = 44.38, *P* < 0.001]. Overall, the data from experiment 3 (with three pairs of brackets) extend our previous findings to longer and more demanding sequences, confirming that crows can apply recursive capabilities to solve the task with novel embedded pairings. While we cannot exclude that swap trials may be represented as (nonrecursive) ordinal lists (*27*), ordinal list information cannot account for the high performance in joint categories that are characterized by conflicting ordinal positions of the brackets. The crow data, although different from each other, are not explained by a simple ordinal model (fig. S4). The fact that the crows were significantly above chance on the joint trials is compelling evidence that they used a recursive strategy.

**Fig. 4.**
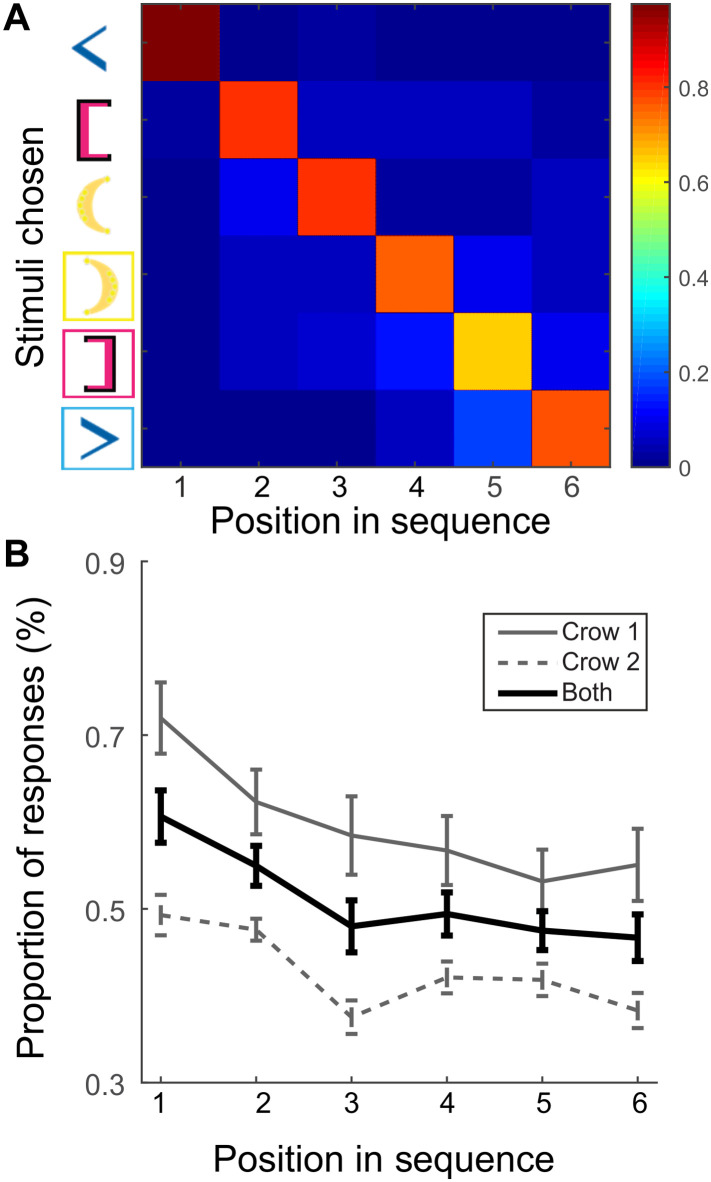
Relationship between bracket position and performance in three-pair bracket sequences. (**A**) Example response matrix showing average choice proportion across transfer trials with the *x* axis being the position in the sequence and the *y* axis being the bracket stimulus chosen. Stimuli are ordered on the basis of the highest proportion for that position in which the bracket was chosen. (**B**) The highest choice proportion as a function of bracket ordinal position in a sequence [diagonal values of (A) are plotted].

Even with recursive understanding, longer sequences put more demand on working memory because the order of more relationships needs to be maintained in mind. This interaction with working memory constraints was also previously investigated (*14*); children who produced more center-embedded sequences were more likely to have a higher working memory capacity. We hypothesized that the crows’ decrease in overall performance when moving from two pairs to three pairs of brackets stemmed from increased working memory demands. We therefore explored the relationship between the length of the sequence and the performance on the bracket task. In the three-pair experiment, the crows were trained to select six stimuli one after another. Because all responses in transfer trials were equally rewarded, we were able to collect all the responses for each bracket stimulus in each position of the sequence to create a response matrix. The choice probabilities of the crows for each bracket in the sequence were depicted as confusion matrices (example in Fig. 4A); the high probabilities along the diagonal reflect the crows’ reliability in selecting particular bracket stimuli during these transfer trials. In addition, the decreasing choice probabilities toward the end of the sequence indicate that crows become more variable in which bracket they select. We averaged the proportions of a single bracket selected at each position on the sequence across all transfer trial lists (Fig. 4B). We found a negative relationship between the highest proportion of responses and increasing position in the sequence (ρ = −0.54, one-sample *t* test against the null: *t* = −9.57, *P* < 0.001). This correlation suggests that the crows memorize, albeit imperfectly, which stimuli they previously selected; they become less consistent toward the end of the sequence, and this holds true across all possible transfer lists.

## DISCUSSION

### General

Our results show that carrion crows exhibit the ability to parse and generate center-embedded recursive sequences. This ability is remarkable, given that birds diverged from the primate lineage more than 320 million years ago and consequently have a distinct and independently evolved telencephalon (*28*). The crow data can be directly compared to those of human groups varying in age, culture, and educational experience in addition to macaque monkeys because we adopted the same task procedures from a previous study on recursive sequence processing using bracket stimuli (*14*). Crows perform on par with 3- to 4-year-old children (*14*), producing around 40% center-embedded responses, which is significantly above the proportion of crossed responses upon first exposure to the bracket lists. In this sense, they outperformed the macaque monkeys who initially preferred to use an ordinal strategy (*29*), producing equal proportions of center-embedded and crossed responses. With additional training using the same experimental design, the monkeys started using the abstract hierarchical structure. They were also able to generalize the center-embedded structure to completely novel stimuli in a subsequent experiment (*14*). These experiments demonstrate that it is not a capacity difference with the macaques but rather a divergence in the requisite amount of experience with these structures. So, the success of the crows upon the first exposure encourages us to say that crows join humans in their capacity to extract the underlying recursive structure given few exemplars during training. While a sample size of two is not enough to infer that any crow in the population may generate center-embedded recursive sequences, we present a “proof of existence” showing that this cognitive capacity is, in principle, within the reach of carrion crows.

### Increased depth of embeddings

With only two pairs of brackets, it cannot be excluded that the crows could have used alternative strategies rather than recursion to solve the task in transfer trials (*30*). Because the training portion of the bracket task does not explicitly require representation and reproduction of the center-embedded structure of those lists, it is possible that animals may exploit other nonrecursive strategies. Upon the first exposure for monkeys, they did not make significantly more center-embedded to crossed responses, suggesting that they adopted an ordinal strategy where they chose brackets on the basis of their closest distance from the training positions. Upon further training, the monkeys then started to produce significantly more center-embedded responses than crossed responses, suggesting that they might have needed more experience to infer the complex center-embedded structure of the bracket lists (*14*).

However, others have put forth that this performance could be alternatively explained by the monkeys having adopted a combination of ordinal and associative strategies with a heavier priority on the ordinal position when selecting brackets. After selecting either combination of open brackets “ ( [ ” or “ [ ( ”, the monkeys might have picked up that certain brackets were paired systematically such that “ [ ” was always followed by “ ] ” and “ ( ” by “ ) ”, with a transition probability of one. This complicated junction of multiple strategies becomes much less probable with increasing the length of the sequence. For two pairs, there are two possible response sequences that would be considered “center-embedded” out of 24 possible responses; this results in a chance level of ~8%. However, with three pairs, the number of response sequences is markedly expanded with six possible center-embedded response sequences out of 720 possibilities; this results in a chance level of less than 1%.

This was a major motivation for us to challenge the crows to an increased depth of three bracket pairs, which was yet to be tested. This expansion is not trivial because even in humans, most sentences do not exceed two levels in center embedding (*31*). Yet, despite the substantially increased demand, both crows were still able to continuously perform the three–bracket pair sequence production task in novel transfer trials in both swap and joint trial types. In particular with the joint trials, the alternative multiplexed ordinal-associative strategy would not be feasible. For example, with a transfer list consisting of the outer pairs of training lists 1 and 2 and the middle pair of either training list, none of the bracket pairs have a known transition of 1 between them (e.g., none of them are inner pairs), making it unclear how these stimuli would be ordered to produce a center-embedded structure (see fig. S4 for additional examples).

It is important to note that recursive abilities do not exist in a vacuum independent of other cognitive abilities. While these experiments reveal that the crows induce the recursive structure of the sequences and not just the ordinality of individual elements during training, it is possible that the hierarchical/recursive capacities do contain an ordinal component, as indicated by the crows’ tendency to start sequences with specific bracket types (fig. S1). In addition, working memory capacities are challenged in such tasks because more bracket identities and positions must be kept in mind during production of the longer sequences. In other words, increasing the depth of the embedding correspondingly increases working memory loads. Thus, the complexity of center-embedded sequences increases as a function of the depth of embeddings (*32*). As expected, the performance of the crows on the three–bracket pair sequences, albeit highly significant, was lower compared to the two-pair sequences. This pattern is distinct from a human study (*9*)—which used the same spoken syllable stimuli as in a study with tamarins (*4*)—where they found that performance improved with an increase in sequence length. This patterning suggests that subjects in this study were not processing the audio sequences as center-embedded structures but rather discriminating between sound category transitions and alternations. One strength of the current stimulus set of colored bracket pairs is that the open and close directions did not drive the crows to also adopt a transition/alternation strategy.

### Spontaneously generalizing to deeper center-embedded structures

It has been suggested (*30*, *33*) that the pivotal test for recursive understanding would be to present deeper recursion nesting during transfer trials than those trained on (i.e., trained on a fixed two–bracket pair depth and tested on unseen lists with three pairs). However, we shied away from this as even human children struggle with spontaneously generalizing to deeper center-embedded structures. For illustrative purposes of these difficulties, we discuss another milestone in acquiring an understanding of symbolic systems—when children learn than number symbols are embedded in integer lists (*34*). By using recursive rules, humans can hypothetically continue counting indefinitely. However, when learning number words, children first grasp the meaning for “one” and cannot immediately generalize to larger counts. Months later, they pick up the meaning for “two,” then “three,” and “four.” Only after this stage are they able to accurately count and generate sets for even larger numbers (*35*). Children recognize the successor function, thus mapping the relationship between the numerals and values, only with multiple presentations at different depths. Given these challenges even in children, we opted to train the crows with two pairs and three pairs before testing them with novel stimulus combinations of the same depth within each experiment. It remains unclear how an animal would know how to respond to such deeper recursive nesting even if they knew, in principle, recursion. Multiple training stages with two pairs, then three pairs, and even four pairs before testing on higher–pair number sequences are untenable because of the steep increase in working memory demands.

### Impact of working memory demands on recursive processing

Parsing center-embedded recursive structures requires working memory devices (e.g., stack of pointers) to keep track of where in the sequence one is at after a previous embedded element has been completed. In the previous study (*14*), individual differences in children’s working memory was positively correlated with their ability to generate center-embedded sequences. With a smaller sample size, we were unable to perform a similar correlation. Instead, we approached working memory constraints in an orthogonal way by examining the relationship between position in the sequence and response variability. With longer sequences, it becomes expansively more difficult to keep track of which bracket stimuli were previously selected and which ones are yet to be selected. When we correlated the response variability with the position along the length of a three-pair bracket sequence, we found that crows’ consistency suffered further down the sequence. We interpret this behavioral pattern as a reflection of working memory constraints rather than a lapse of recursive understanding in crows per se. In humans, short-term memory for serial order shows equivalent performance effects as those we saw in the crows. When humans are required to recall predefined verbal sequences in forward order, a monotonic decrease in recall accuracy extending from the first position onward is noted [the primacy effect; (*36*)]. This effect was present in crows performing on the three-pair bracket sequences. Although the recall of predefined sequences in the human study is not identical to the self-generated internal ordering of sequences required from crows in the current study, the similarity of the effects still suggests that recursive processing in our crows is progressively limited by working memory capacity with increasing sequence length and depth of embedding.

### Evolution of recursive processing

With this collection of experiments, we can add corvids to the group of animals that can parse minimally recursive patterns. Recursive parsing has been nominated as a complex computation that might delineate human language, one of several symbol systems, from the communication systems of all other animal species (*37*–*38*). Exploring recursion, recursive-adjacent processing, and other putative syntactic rules in diverse animal species (*39*–*43*) is critical in delineating the associated evolutionary implications. For example, corvids having a markedly different telencephalic brain architecture as compared to primates (*44*–*45*); they lack a layered neocortex, which is thus not strictly necessary to support recursive processing. Our finding also further suggests corvid songbirds to be a promising candidate to study the mechanisms underlying complex communicative cognition in nonhuman animals.

## MATERIALS AND METHODS

### Participants

Two male carrion crows (*C. corone*; bird 1: 8 years old and bird 2: 8 years old) participated in this study. They were hand-raised and housed socially in large indoor aviaries. The crows were on a controlled feeding protocol during the training periods. Daily food was given via rewards during training sessions and was supplemented afterward if necessary. Water was provided ad libitum in the aviary and during experiments. All procedures were carried out in accordance with European law and the *Guidelines for the Care and Use of Laboratory Animals* from the National Institutes of Health and were approved by the responsible national authorities (Regierungspräsidium Tübingen).

### Experimental design

The crows were trained and tested in a darkened operant conditioning chamber in front of a touchscreen monitor (3M Microtouch, 38.1 cm, 60-Hz refresh rate). Visual stimuli were presented using the CORTEX program (National Institute of Mental Health, USA) with a viewing distance of 14 cm. Rewards (mealworms or bird seed pellets) were delivered via an automated feeder after correctly performed trials.

To begin a trial, crows positioned their heads (with an attached reflective foil) under an infrared light barrier. Subsequent steps are adopted from the macaque monkey training procedures (*14*). Stimuli (brackets) appeared in random configurations on the monitor. The crows were tasked with pecking each stimulus in the correct order within 10 s. After each correct peck, the selected stimulus would flash and give an auditory cue that the touch was registered. These registered items would remain on the screen after pecking but were unable to be chosen again for 2 s to prevent continuous pecking. If a stimulus was incorrectly chosen, the crow was presented with auditory feedback (buzzer) and a 2-s time-out screen. If all stimuli were correctly chosen thus completing the sequence, the crows were rewarded with positive auditory feedback and food reward. There was then a 2-s intertrial interval before the next trial could be initiated. During transfer trials, every trial was rewarded so long as all stimuli were selected in any sequence. Trials in which the crow prompted the bracket array but did not select any stimulus within the allowed time were coded as misses. Trials in which the crow selected fewer than the total number of stimuli shown were coded as incompletes. Misses and incompletes were not analyzed further.

### Sequence training phase

The participating crows did not have prior experience with sequencing tasks and were mostly familiar with delayed-match-to-sample and two-alternative-choice tasks. This is in contrast with the macaques (*14*), who were all familiar with the general structure and demands of sequencing tasks. For our crows, we first had to train them to peck in a sequence using nontask-relevant photographic stimuli. First, a flower photo appeared on the screen and had to be pecked. The next day, a car photo appeared on the screen with the flower and had to be selected after a peck to the flower. After crows could successfully complete sequences with 70% accuracy, a new stimulus was introduced and appended to the end of the previous sequence in the subsequent session. This continued until a fourth photo sequence was reliably selected (photo sequence: flower ➔car➔tree➔bird). After reaching a criterion of more than 70% performance over two consecutive sessions, crows were moved to the bracket recursion sequencing task (*14*).

### Experiment 1

The crows were trained on two center-embedded lists (Fig. 1B) before proceeding onto test sessions with transfer trials. Each list consisted of two bracket pairs (i.e., four stimuli), “{ ( ) }” and “{ [ ] }” with the “{ }” in purple, “( )” in orange, and “[ ]” in pink. These bracket stimuli have the advantages of inherently cuing pair relationships via the type and color of the brackets as well as the order via open/close positions. For each list, the two pairs were introduced at the same time, i.e., there was no training phase where only one pair was presented.

In addition to the direction of the brackets, a border was added to the closed brackets to better define the order. These perceptual cues also allowed us to bypass a one-pair (two-item) training phase to learn what base pairs belonged together and in which order. This procedure circumvents the dependence on associative strategies promoted by one-pair training. Subjects were first presented with training list 1 “{ [ ] }” until they met criterion, which we defined as 70% correct for two consecutive days. They were then moved onto the training list 2 “{ ( ) }” until the same criterion was reached. Crows took, on average, 8 days to reach criterion per list (crow 1: 6 days and crow 2: 10 days). For this first experiment, the crows required more trials to reach the criterion on both training lists than the monkeys (*14*); this difference is likely caused by the monkeys being familiar with the general structure and demands of sequencing tasks before the experiments unlike the crows.

Following completion of the training phase, on two consecutive days, the crows were confronted with the test sessions containing transfer trials. In these sessions, the two training lists were intermixed to make up 90% of all trials. The remaining 10% were transfer trials in which the two middle bracket pairs “[ ]” and “( )” of the training lists were presented together. Notably, these test trials where the first instance these pairs were presented to the crows together. These trials were rewarded for any sequence so long as the crows pecked all four stimuli. Transfer trials that contained a repeated peck of the same bracket was allowed but did not occur. For experiment 1, crow 1 completed 106 responses out of 130 total transfer trials, and crow 2 completed 105 responses out of 120 total transfer trials.

Similar to humans and monkeys, many of the crow responses could not be classified as center-embedded, tail-embedded, or crossed; around 36% of responses did not fall cleanly into one of the above categories (e.g., the bars in Fig. 1D, see fig. S1). However, for direct comparison to previous work, we focused our analyses on the three main response types: tail-embedded “( )[ ]”, crossed “( [ ) ]”, and center-embedded “( [ ] )”.

### Experiment 2

To test whether crows were sensitive to the ordering of the bracket stimuli from training, we presented them with two additional center-embedded lists (fig. S2). These lists involved novel bracket pairs that were presented with the same general procedure as experiment 1. Brackets here were varied in types, fonts, and colors with “< >” in blue, “[ ]” in green, and “( )” in yellow. The difference here was that the outside brackets were not held constant while the inside brackets were swapped. Instead, we had one bracket pair “[ ]” present in both the outside and inside positions. Here, training list 1 was “< [ ] >”. Training list 2 was “[ ( ) ]”. The crows took, on average, 4 days to reach criterion per list (crow 1: 3 days and crow 2: 5 days).

After completion of training, test sessions were presented where 90% of trials that consisted of the two training lists were intermixed and the remaining 10% consisted of novel transfer trials. The transfer trials were the previously never presented together bracket pairs—< > and ( ). These trials were rewarded for any sequence so long as the crows pecked all four stimuli. For experiment 2, crow 1 completed 129 responses out of 133 total transfer trials, and crow 2 completed 132 responses out of 150 total transfer trials. We examined the response patterns (fig. S2) for the transfer trials. In addition to examining the proportion of tail-embedded, crossed, and center-embedded responses, we also examined the order of the selected bracket stimuli in the center-embedded responses.

### Experiment 3

To test whether the crows were able to perform recursive sequence generation at a deeper level, we increased the depth from two to three pairs for the final experiment. Training stimuli consisted of the three unique pairs used in experiment 1 and experiment 2. Training list 1 was {_purple_ [_pink_ (_orange_) _orange_] _pink_}_purple_. Training list 2 was <_blue_ [_green_ (_yellow_)_yellow_]_green_ > _blue_. Criterion for this experiment was modified given that chance value for producing a correct sequence of six items is much lower than that of four items (<1% versus ~4%). Crows were required to perform above 60% correct for 2 consecutive days. They took, on average, 15 days to reach criterion per list (crow 1: 11 days and crow 2: 19 days). As with the previous experiments, crows were presented with test sessions after successful conclusion of training lists. Test sessions consisted of 90% intermixed training list trials combined with 10% new transfer trials.

Because many combinations of transfer trials lists could be made (fig. S3), we split the lists across multiple days of testing depending on which original pair position was modified. On the first day, we presented six transfer lists in which the inner most pairs of the list were manipulated. Then, on the second day, six transfer lists manipulating the middle pairs were presented. On the third day, we presented six transfer lists in which the outer most pairs of the list were manipulated. This cycle continued for a total of 12 days until each target pair manipulation was repeated four times. Of the six lists each day (Fig. 3C and fig. S3), two lists were classified as swap trial types whereby pairs of the same position of the training lists were interchanged (i.e., the yellow inner pair of training list 2 was swapped with the orange inner pair of training list 1). The remaining four lists were categorized into joint trial types whereby pairs of the same position were presented together with a single other pair (i.e., the yellow inner pair of list 2 was combined with the orange inner pair and the purple outer pair of list 1). All transfer trials were rewarded regardless of the order in which the stimuli were responded to (i.e., 100% chance of reward). For experiment 3, due to the increased number of pairs and thus transfer lists, crows 1 and 2 were presented with 878 and 802 transfer trials, respectively. Of those, they completed 718 and 625 full six-stimulus sequence responses, respectively.

### Statistical analyses

Responses to transfer trials were identified and organized by the response types. Only trials in which all six stimuli were chosen were used in the following analyses; we excluded miss and incomplete trials. Miss trials occurred when the bracket stimuli appeared on the screen, but the crow did not respond to any stimulus. Incomplete trials occurred when the bird responded to at least one but not all stimuli on the screen.

All analyses were done in MATLAB version 2018,2020 and R. We used binomial tests to test whether different responses were different from chance. To test whether there was a difference in the frequency of responses, we used chi-square tests for independence and goodness of fit with Yates’ continuity correction. All tests were two-tailed. Numbers, test statistics, and *P* values are reported. Alpha was set to α = 0.05.

We generated a response matrix for each transfer trial list to examine the relationship between bracket position and reliability of the responses. Because each column represents a position in the sequence, we isolated the highest proportion in each column and then calculated a correlation coefficient (MATLAB function: corrcoef). We got a total of 36 coefficients (18 different transfer trial lists, two crows), and we tested these values against the null with a one-sample *t* test.

## Notes

View/request a protocol for this paper from *Bio-protocol*.
