# Supplementary material for: Recursive sequence generation in crows
Source: Sci Adv. 2022 Nov 2;8(44):eabq3356. doi: 10.1126/sciadv.abq3356 (PMC9629703; doi:10.1126/sciadv.abq3356)
Supplement: Supplementary file 1 — Figs. S1 to S4 Tables S1 and S2 [file sciadv.abq3356_sm.pdf]

Science Advances
AAAS

Supplementary Materials for

Recursive sequence generation in crows

Diana A. Liao *et al.*

Corresponding author: Diana A. Liao, diana.a.liao@gmail.com; Andreas Nieder, andreas.nieder@uni-tuebingen.de

Sci. Adv. **8**, eabq3356 (2022)
DOI: 10.1126/sciadv.abq3356

This PDF file includes:

Figs. S1 to S4
Tables S1 and S2

Supplementary Figures

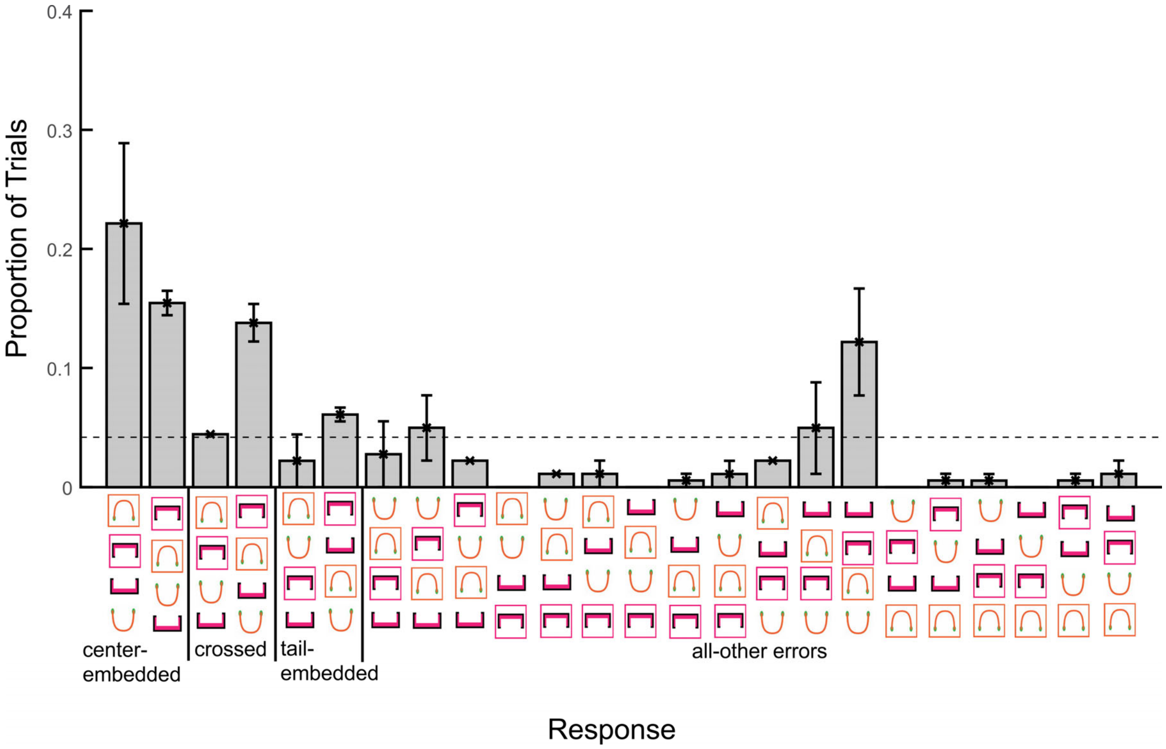

Figure S1: The proportion of each possible response (n = 24) for two pair transfer trials for Experiment 1. Error bars represent the standard error of the mean (SEM) across both crows.

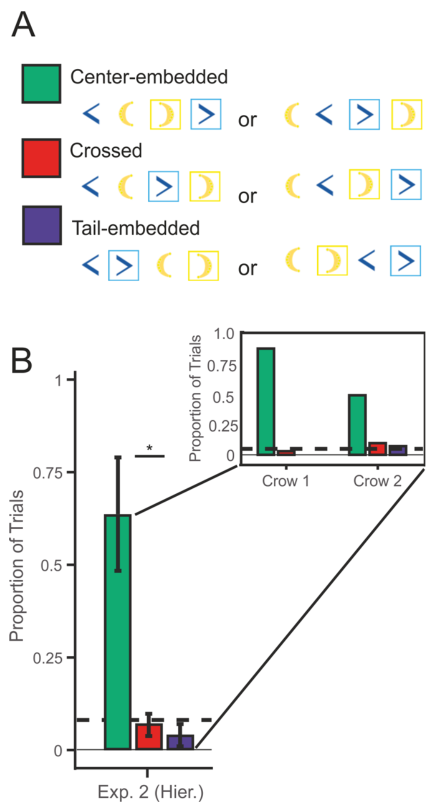

Figure S2: Responses to hierarchical ordering of bracket stimuli. (A) Response types during transfer trials, which were rewarded regardless of the selected order. **(B)** Proportion of response types produced. Inset display results for each bird. Error bars represent the SEM of the population, * represents a significant difference ($p < 0.05$) between the proportion of center-embedded and crossed responses.

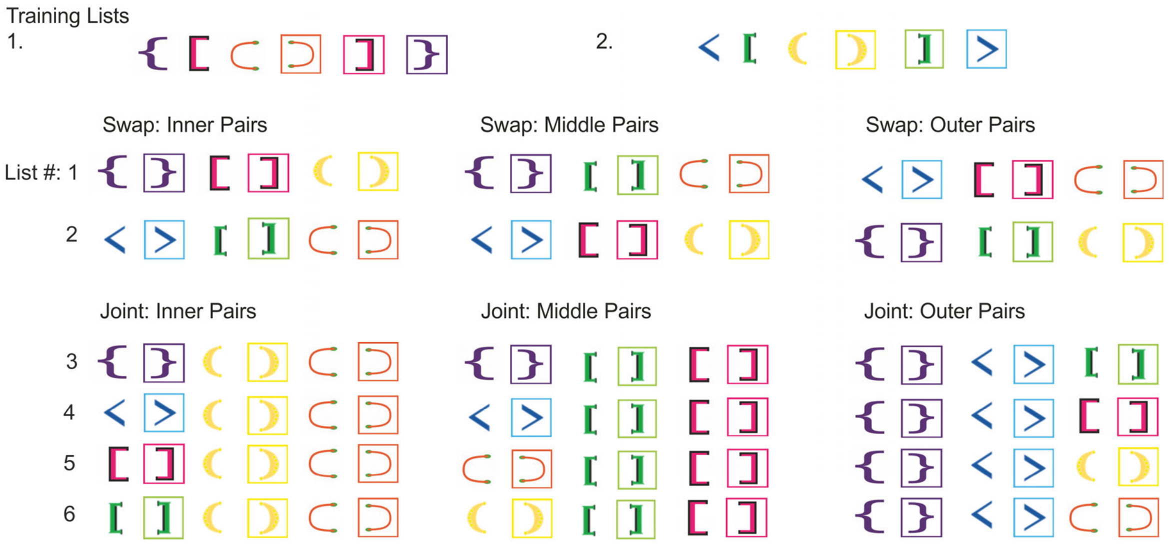

Figure S3: All combinations for three pair transfer trials. With two training lists consisting of three pairs, many different transfer trial lists (composed of novel bracket combinations) can be created. There are three pair positions for three pair lists: inner, middle, and outer. Each column refers to the position in the list that is altered. For each position, six new transfer trial lists were constructed and separated into two categories. Two of the lists are called “swap” trials whereby the corresponding pair from the other list is swapped in. The remaining four lists are “joint” trials whereby pairs belonging in the same position are presented together with the remaining stimuli.

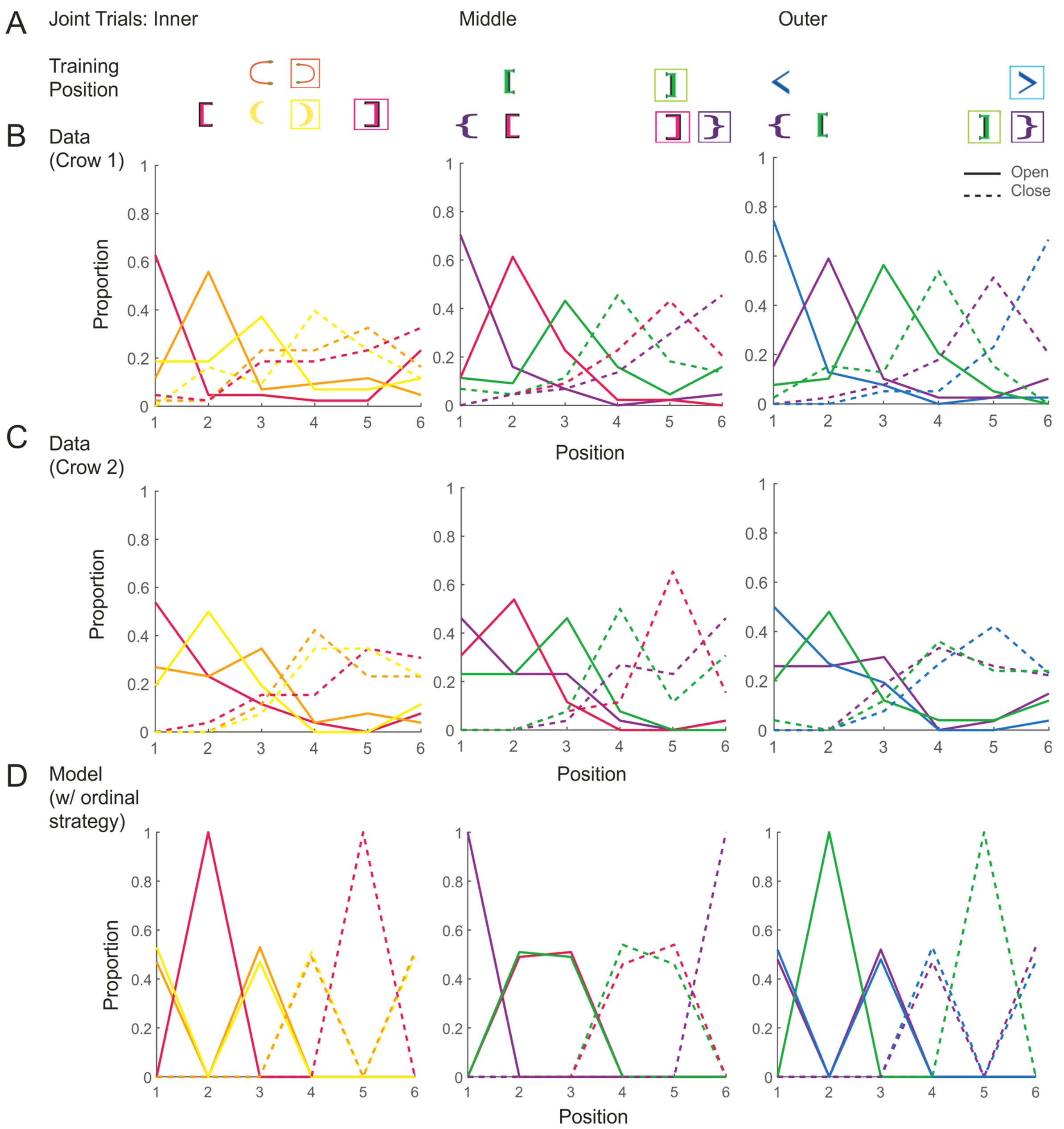

Figure S4: The crows' responses proportions in joint trials compared to an ordinal model prediction. A) Brackets from exemplar joint transfer lists (1 each from inner, middle, and outer pairs) placed where their training position was. **B,C**) The positions of where these brackets were chosen by crow 1 (**B**) and crow 2 (**C**). **D**) Performance of a simple model that follows ordinality. Given the brackets in each particular transfer list, the model compares the queried positions with the previously trained positions. It selects the bracket that matches to that during training to fill the position. If there are two matches, the model randomly selects one of the acceptable brackets. The remaining brackets are placed in the remaining positions. For example, in the outer transfer list (third column above), there are two outer pairs and one middle pair. The model selects either outer-pair open bracket randomly. Then for position 2, it selects the middle pair open bracket. For positions 3, the remaining open bracket is selected. Interestingly, there are no inner pairs presented here so there is no consistent transition between positions 3 and 4. A similar order would follow for positions 4-6 with the set of closed brackets.

Supplementary Tables

Supplementary Table 1: Number of total training trials per training list to reach criterion.

Crow	Exp. 1		Exp. 2		Exp 3	
	**List 1**	**List 2**	**List 1**	**List 2**	**List 1**	**List 2**
**1**	6150	1239	1723	1888	13071	3140
**2**	6308	2404	1812	2669	15277	5317

Supplementary Table 2: Number of center-embedded, crossed, tail-embedded and other responses for each crow at the beginning (first 20 transfer trials) versus the end (last 20 transfer trials) of test sessions.

Crow	Response Type	Experiment 1		Experiment 2		Experiment 3	
		Beginning	End	Beginning	End	Beginning	End
1	Center	8	13	19	18	11	10
	Cross	6	0	0	0	0	0
	Tail	0	1	1	0	0	0
	Other	6	6	0	2	9	10
2	Center	5	8	14	13	4	10
	Cross	5	2	3	0	0	0
	Tail	1	2	0	0	0	0
	Other	9	8	3	7	16	10
